# 2,3,6,7-Tetra­bromo-9-butyl-9*H*-carbazole

**DOI:** 10.1107/S1600536812013761

**Published:** 2012-04-13

**Authors:** J. Josephine Novina, G. Vasuki, Sushil Kumar, K. R. Justin Thomas

**Affiliations:** a Department of Physics, Idhaya College for Women, Kumbakonam-1, India; b Department of Physics, Kunthavai Naachiar Govt. Arts College (W) (Autonomous), Thanjavur-7, India; c Organic Materials Lab, Department of Chemistry, Indian Institute of Technology Roorkee, Roorkee 247 667, India

## Abstract

In he title compound, C_16_H_13_Br_4_N, the carbazole skeleton is nearly planar [maximum deviation = 0.026 (4) Å] and makes a dihedral angle of 73.8 (4)° with the butyl chain. The butyl chain adopts a *trans* conformation. In the crystal, mol­ecules are linked by π–π stacking inter­actions [centroid–centroid distance = 3.559 (2) Å].

## Related literature

For general background to carbazole derivatives, see: Uludağ *et al.* (2011 ▶); Zuluaga *et al.* (2011 ▶). For their biological activity, see: Kubicki *et al.* (2007 ▶); Lohier *et al.* (2010 ▶) and for their applications, see: Thomas *et al.* (2001 ▶); Tsuboyama *et al.* (2003 ▶). For related structures, see: Ergün *et al.* (2010 ▶); Saeed *et al.* (2010 ▶); Chen *et al.* (2009 ▶); Gagnon & Laliberté (2008 ▶). For standard bond lengths, see: Allen *et al.* (1987 ▶).
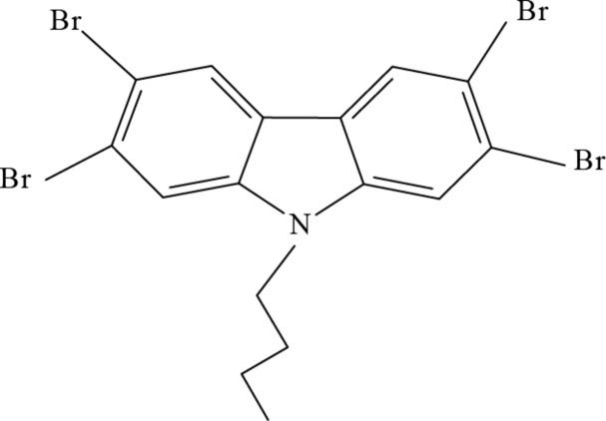


## Experimental

### 

#### Crystal data

C_16_H_13_Br_4_N
*M* *_r_* = 538.91
Triclinic, 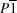

*a* = 8.7127 (4) Å
*b* = 9.5712 (4) Å
*c* = 11.3379 (5) Å
α = 87.225 (2)°
β = 72.014 (2)°
γ = 67.673 (2)°
*V* = 829.30 (6) Å^3^
*Z* = 2
Mo *K*α radiation
μ = 9.70 mm^−1^
*T* = 293 K
0.30 × 0.25 × 0.20 mm

#### Data collection

Bruker Kappa APEXII CCD diffractometer
Absorption correction: multi-scan (*SADABS*; Bruker, 2004 ▶) *T*_min_ = 0.159, *T*_max_ = 0.247
18599 measured reflections
3816 independent reflections
2739 reflections with *I* > 2σ(*I*)
*R*_int_ = 0.050

#### Refinement

*R*[*F* ^2^ > 2σ(*F* ^2^)] = 0.038
*wR*(*F* ^2^) = 0.092
*S* = 1.05
3816 reflections
190 parameters
H-atom parameters constrained
Δρ_max_ = 0.45 e Å^−3^
Δρ_min_ = −1.03 e Å^−3^

### 

Data collection: *APEX2* (Bruker, 2004 ▶); cell refinement: *APEX2* and *SAINT* (Bruker, 2004 ▶); data reduction: *SAINT* and *XPREP* (Bruker, 2004 ▶); program(s) used to solve structure: *SIR92* (Altomare *et al.*, 1994 ▶); program(s) used to refine structure: *SHELXL97* (Sheldrick, 2008 ▶); molecular graphics: *ORTEP-3* (Farrugia, 1997 ▶) and *Mercury* (Macrae *et al.*, 2008 ▶); software used to prepare material for publication: *PLATON* (Spek, 2009 ▶).

## Supplementary Material

Crystal structure: contains datablock(s) I, global. DOI: 10.1107/S1600536812013761/bx2400sup1.cif

Structure factors: contains datablock(s) I. DOI: 10.1107/S1600536812013761/bx2400Isup2.hkl

Supplementary material file. DOI: 10.1107/S1600536812013761/bx2400Isup3.cml

Additional supplementary materials:  crystallographic information; 3D view; checkCIF report

## Figures and Tables

**Fig. 1.**
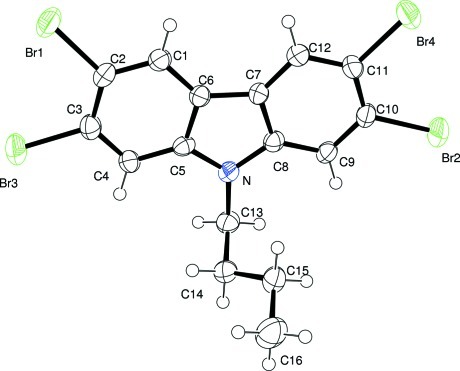
The molecular structure of the title compound, with atom numbering and displacement ellipsoids drawn at the 50% probability level.

**Fig. 2.**
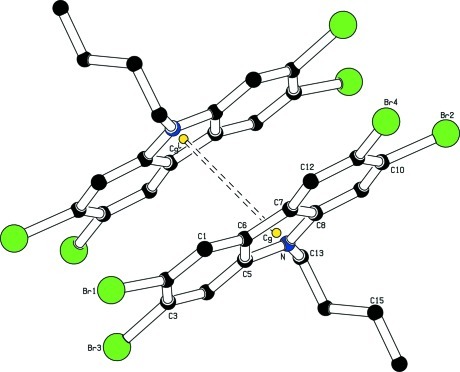
π–π stacking interactions. Symmetry code: i: -x,1-y,-z

## supplementary crystallographic information

### Comment

Carbazole and its derivatives have become quite attractive compounds owing to their applications in pharmacy and molecular electronics. It has been reported that carbazole derivatives possess various biological activities such as antitumor, antioxidative, anti-inflammatory, antimutagenic, anticancer, antibacterial and antifungal activities (Kubicki *et al.*, 2007; Lohier *et al.*, 2010). They also have an important role in the synthesis of indole alkaloids. (Ergün *et al.*, 2010). On the other hand, carbazole and its derivatives are very attractive compounds because of their charge transporting (Saeed *et al.*, 2010), thermal and emission properties. Due to this they are also considered as potential candidates for application in electronic devices, such as organic light-emitting diodes (OLEDs) (Thomas *et al.*, 2001; Kubicki *et al.*, 2007), thin-film transistors and solar cells. Carbazole-based compounds have been widely utilized as the host material for efficient green and red phosphorescent organic light-emitting diodes (PhOLEDs) due to their favorable triplet energies. (Tsuboyama, *et al.*, 2003). The title compound (I), consists of a carbazole skeleton substituted with four bromides at the 2,3,6 and 7 positions and a *n*-butyl group attached to atom N (Fig.1), where the bond lengths(Allen *et al.*, 1987) and angles are within normal ranges, and generally agree with those found in related structures, 9-Butyl-9*H*-cabazole [Chen *et al.*, 2009], 1,1'-(9-Octyl-9*H*-cabazole-3,6-diyl)-diethanone [Saeed *et al.*, 2010], 2,7-Dibromo-9-octyl-9*H*-cabazole [Gagnon and Laliberté, 2008]. The dihedral angle formed by the least-square planes of the carbazole system and butyl chain is 73.8 (4)°. An examination of the deviations from the least-squares planes through individual rings show that ring A(C1—C6), B(C5—C8/N) and C(C7—C12) are planar [with a maximum deviation of 0.026 (4) Å for atom C3] with dihedral angle of A/B=1.51 (29)°, A/C=2.87 (26)° and B/C=1.70 (29)° are in close agreement with the values that observed in similar structures 9-(4-Nitrophenylsulfonyl)-9*H*-carbazole [Uludağ *et al.*, 2011], 11-Butyl-3-methoxy-11*H*-benzo[*a*]-carbazole [Ergün *et al.*, 2010]. Specifically,the bonds labelled here as C1—C2, C3—C4, C9—10, C11—C12 are shorest bond in the six membered rings, while the C6—C7 bond is the longest C—C bond in the carbazole unit (Zuluaga *et al.*, 2011). The valence angle centred on C13, C14 and C15 are less than 120° [113.4 (3)°, 114.6 (3)° and 113.6 (3)°, respectively] and consequently, the C13—C14 and C15—C16 bonds deviate from the symmetry axis of the central ring of the carbazole system. The torsion angle C6—C5—N—C13= -179.65 (27)° indicates that the butyl chain adopts a *trans* conformation with respect to C5—N bond. In the crystal, the molecules are linked by π -π stacking interactions.( Cg: N/C5-C8); with Cg–Cg^i^ 3.559 (2) Å, α=0°).

### Experimental

The title compound was synthesized by treating 2,7-dibromo-9-butyl-9*H*-carbazole with two equivalents of *N*-bromosuccinimide in dimethylformamide for 24 hrs. After completion of the reaction, the title compound was obtained by filtration after pouring the reaction mixture into ice-cold water. It was recrystallized from dichloromethane/hexane mixture [Yield: 71%].

### Refinement

All the H atoms were poistioned geometrically and treated as riding on their parent atoms: *C*—H = 0.93, 0.96 and 0.97 Å for CH, CH_3 _and CH_2_ H atoms,respectively, and refined using riding model with *U*_iso_(H) =Kx*U*_eq_(parent C-atom), where K=1.5 for CH_3_ H atoms and K=1.2 for all other H-atoms.

### Crystal data

**Table:**

C_16_H_13_Br_4_N	*Z* = 2
*M**_r_* = 538.91	*F*(000) = 512
Triclinic, *P*1	*D*_x_ = 2.158 Mg m^−^^3^
*a* = 8.7127 (4) Å	Mo *K*α radiation, λ = 0.71073 Å
*b* = 9.5712 (4) Å	Cell parameters from 3816 reflections
*c* = 11.3379 (5) Å	θ = 2.3–27.5°
α = 87.225 (2)°	µ = 9.70 mm^−^^1^
β = 72.014 (2)°	*T* = 293 K
γ = 67.673 (2)°	Block, colourless
*V* = 829.30 (6) Å^3^	0.30 × 0.25 × 0.20 mm

### Data collection

**Table:**

Bruker Kappa APEXII CCD diffractometer	3816 independent reflections
Radiation source: fine-focus sealed tube	2739 reflections with *I* > 2σ(*I*)
Graphite monochromator	*R*_int_ = 0.050
ω and φ scan	θ_max_ = 27.5°, θ_min_ = 2.3°
Absorption correction: multi-scan (*SADABS*; Bruker, 2004)	*h* = −11→11
*T*_min_ = 0.159, *T*_max_ = 0.247	*k* = −12→12
18599 measured reflections	*l* = −14→14

### Refinement

**Table:**

Refinement on *F*^2^	Primary atom site location: structure-invariant direct methods
Least-squares matrix: full	Secondary atom site location: difference Fourier map
*R*[*F*^2^ > 2σ(*F*^2^)] = 0.038	Hydrogen site location: inferred from neighbouring sites
*wR*(*F*^2^) = 0.092	H-atom parameters constrained
*S* = 1.05	*w* = 1/[σ^2^(*F*_o_^2^) + (0.0467*P*)^2^ + 0.1503*P*] where *P* = (*F*_o_^2^ + 2*F*_c_^2^)/3
3816 reflections	(Δ/σ)_max_ < 0.001
190 parameters	Δρ_max_ = 0.45 e Å^−^^3^
0 restraints	Δρ_min_ = −1.03 e Å^−^^3^

### Special details

**Table:**

Geometry. All e.s.d.'s (except the e.s.d. in the dihedral angle between two l.s. planes) are estimated using the full covariance matrix. The cell e.s.d.'s are taken into account individually in the estimation of e.s.d.'s in distances, angles and torsion angles; correlations between e.s.d.'s in cell parameters are only used when they are defined by crystal symmetry. An approximate (isotropic) treatment of cell e.s.d.'s is used for estimating e.s.d.'s involving l.s. planes.
Refinement. Refinement of *F*^2^ against ALL reflections. The weighted *R*-factor *wR* and goodness of fit *S* are based on *F*^2^, conventional *R*-factors *R* are based on *F*, with *F* set to zero for negative *F*^2^. The threshold expression of *F*^2^ > σ(*F*^2^) is used only for calculating *R*-factors(gt) *etc*. and is not relevant to the choice of reflections for refinement. *R*-factors based on *F*^2^ are statistically about twice as large as those based on *F*, and *R*- factors based on ALL data will be even larger.

### Fractional atomic coordinates and isotropic or equivalent isotropic displacement parameters (Å^2^)

**Table:**

	*x*	*y*	*z*	*U*_iso_*/*U*_eq_	
C11	−0.0404 (4)	0.8421 (4)	−0.2274 (3)	0.0339 (8)	
C13	0.2397 (4)	0.2502 (4)	−0.2560 (3)	0.0367 (9)	
H13A	0.1541	0.2705	−0.2992	0.044*	
H13B	0.2257	0.1748	−0.1979	0.044*	
C14	0.4196 (5)	0.1866 (4)	−0.3490 (3)	0.0399 (9)	
H14A	0.4358	0.0914	−0.3874	0.048*	
H14B	0.5046	0.1647	−0.3051	0.048*	
C15	0.4566 (5)	0.2883 (4)	−0.4502 (4)	0.0399 (9)	
H15A	0.3721	0.3101	−0.4946	0.048*	
H15B	0.4406	0.3836	−0.4121	0.048*	
C16	0.6373 (6)	0.2225 (5)	−0.5420 (4)	0.0609 (12)	
H16A	0.6519	0.2930	−0.6034	0.091*	
H16B	0.6535	0.1295	−0.5819	0.091*	
H16C	0.7221	0.2030	−0.4993	0.091*	
Br4	−0.14241 (6)	1.05229 (4)	−0.23930 (4)	0.05545 (15)	
C1	0.2321 (4)	0.5848 (4)	0.0618 (3)	0.0327 (8)	
H1	0.1932	0.6854	0.0910	0.039*	
C2	0.3235 (4)	0.4702 (4)	0.1212 (3)	0.0335 (8)	
C3	0.3859 (4)	0.3180 (4)	0.0743 (3)	0.0352 (8)	
C4	0.3534 (4)	0.2801 (4)	−0.0269 (3)	0.0322 (8)	
H4	0.3945	0.1794	−0.0565	0.039*	
C5	0.2565 (4)	0.3966 (4)	−0.0850 (3)	0.0291 (8)	
C6	0.1984 (4)	0.5490 (4)	−0.0424 (3)	0.0301 (8)	
C7	0.1094 (4)	0.6384 (3)	−0.1249 (3)	0.0293 (7)	
C8	0.1159 (4)	0.5363 (4)	−0.2117 (3)	0.0296 (8)	
C9	0.0450 (4)	0.5845 (4)	−0.3070 (3)	0.0320 (8)	
H9	0.0498	0.5156	−0.3644	0.038*	
C10	−0.0330 (4)	0.7382 (4)	−0.3138 (3)	0.0329 (8)	
C12	0.0301 (4)	0.7926 (4)	−0.1317 (3)	0.0341 (8)	
H12	0.0241	0.8614	−0.0735	0.041*	
N	0.2045 (4)	0.3899 (3)	−0.1862 (3)	0.0329 (7)	
Br1	0.35722 (6)	0.52290 (5)	0.26623 (4)	0.05058 (14)	
Br2	−0.11763 (6)	0.80537 (5)	−0.44827 (4)	0.05302 (15)	
Br3	0.52215 (6)	0.16149 (5)	0.15025 (4)	0.05527 (15)	

### Atomic displacement parameters (Å^2^)

**Table:**

	*U*^11^	*U*^22^	*U*^33^	*U*^12^	*U*^13^	*U*^23^
C11	0.0365 (19)	0.0273 (17)	0.035 (2)	−0.0103 (14)	−0.0101 (17)	0.0067 (15)
C13	0.048 (2)	0.0304 (18)	0.035 (2)	−0.0173 (16)	−0.0147 (18)	0.0008 (16)
C14	0.051 (2)	0.0328 (19)	0.034 (2)	−0.0114 (16)	−0.0158 (18)	−0.0008 (16)
C15	0.042 (2)	0.045 (2)	0.035 (2)	−0.0163 (16)	−0.0166 (18)	0.0065 (17)
C16	0.050 (3)	0.080 (3)	0.045 (3)	−0.022 (2)	−0.009 (2)	0.005 (2)
Br4	0.0757 (3)	0.0309 (2)	0.0614 (3)	−0.01020 (19)	−0.0373 (2)	0.00839 (19)
C1	0.0383 (19)	0.0312 (18)	0.028 (2)	−0.0129 (15)	−0.0097 (16)	0.0024 (15)
C2	0.0368 (19)	0.043 (2)	0.024 (2)	−0.0184 (15)	−0.0104 (16)	0.0041 (16)
C3	0.039 (2)	0.0337 (19)	0.033 (2)	−0.0146 (15)	−0.0119 (17)	0.0107 (16)
C4	0.0382 (19)	0.0265 (17)	0.030 (2)	−0.0112 (14)	−0.0104 (16)	0.0064 (15)
C5	0.0330 (18)	0.0304 (17)	0.024 (2)	−0.0150 (14)	−0.0054 (15)	0.0014 (14)
C6	0.0320 (18)	0.0294 (17)	0.027 (2)	−0.0111 (13)	−0.0071 (15)	0.0046 (14)
C7	0.0314 (18)	0.0302 (17)	0.025 (2)	−0.0108 (14)	−0.0083 (15)	0.0050 (15)
C8	0.0307 (18)	0.0315 (17)	0.026 (2)	−0.0140 (14)	−0.0061 (15)	0.0039 (15)
C9	0.0376 (19)	0.0355 (19)	0.025 (2)	−0.0147 (15)	−0.0113 (16)	0.0007 (15)
C10	0.0319 (18)	0.041 (2)	0.025 (2)	−0.0122 (15)	−0.0118 (15)	0.0089 (16)
C12	0.0388 (19)	0.0325 (18)	0.029 (2)	−0.0122 (15)	−0.0100 (17)	0.0018 (15)
N	0.0401 (16)	0.0292 (15)	0.0285 (18)	−0.0110 (12)	−0.0124 (14)	0.0014 (12)
Br1	0.0693 (3)	0.0567 (3)	0.0358 (3)	−0.0256 (2)	−0.0287 (2)	0.00626 (19)
Br2	0.0665 (3)	0.0530 (3)	0.0447 (3)	−0.0154 (2)	−0.0350 (2)	0.0109 (2)
Br3	0.0703 (3)	0.0449 (2)	0.0528 (3)	−0.0126 (2)	−0.0362 (2)	0.0171 (2)

### Geometric parameters (Å, º)

**Table:**

C11—C12	1.386 (5)	C1—H1	0.9300
C11—C10	1.400 (5)	C2—C3	1.413 (5)
C11—Br4	1.883 (3)	C2—Br1	1.880 (4)
C13—N	1.466 (4)	C3—C4	1.359 (5)
C13—C14	1.502 (5)	C3—Br3	1.884 (3)
C13—H13A	0.9700	C4—C5	1.396 (5)
C13—H13B	0.9700	C4—H4	0.9300
C14—C15	1.507 (5)	C5—N	1.370 (4)
C14—H14A	0.9700	C5—C6	1.405 (4)
C14—H14B	0.9700	C6—C7	1.440 (5)
C15—C16	1.502 (5)	C7—C12	1.382 (5)
C15—H15A	0.9700	C7—C8	1.394 (5)
C15—H15B	0.9700	C8—C9	1.381 (5)
C16—H16A	0.9600	C8—N	1.386 (4)
C16—H16B	0.9600	C9—C10	1.376 (5)
C16—H16C	0.9600	C9—H9	0.9300
C1—C2	1.374 (5)	C10—Br2	1.876 (4)
C1—C6	1.389 (5)	C12—H12	0.9300
			
C12—C11—C10	120.7 (3)	C3—C2—Br1	122.0 (3)
C12—C11—Br4	118.2 (3)	C4—C3—C2	121.8 (3)
C10—C11—Br4	121.1 (3)	C4—C3—Br3	118.2 (3)
N—C13—C14	113.3 (3)	C2—C3—Br3	120.0 (3)
N—C13—H13A	108.9	C3—C4—C5	118.1 (3)
C14—C13—H13A	108.9	C3—C4—H4	121.0
N—C13—H13B	108.9	C5—C4—H4	121.0
C14—C13—H13B	108.9	N—C5—C4	129.9 (3)
H13A—C13—H13B	107.7	N—C5—C6	109.0 (3)
C13—C14—C15	114.9 (3)	C4—C5—C6	121.0 (3)
C13—C14—H14A	108.6	C1—C6—C5	119.8 (3)
C15—C14—H14A	108.6	C1—C6—C7	133.6 (3)
C13—C14—H14B	108.6	C5—C6—C7	106.6 (3)
C15—C14—H14B	108.6	C12—C7—C8	120.4 (3)
H14A—C14—H14B	107.5	C12—C7—C6	133.1 (3)
C16—C15—C14	113.9 (3)	C8—C7—C6	106.5 (3)
C16—C15—H15A	108.8	C9—C8—N	129.0 (3)
C14—C15—H15A	108.8	C9—C8—C7	121.8 (3)
C16—C15—H15B	108.8	N—C8—C7	109.2 (3)
C14—C15—H15B	108.8	C10—C9—C8	117.6 (3)
H15A—C15—H15B	107.7	C10—C9—H9	121.2
C15—C16—H16A	109.5	C8—C9—H9	121.2
C15—C16—H16B	109.5	C9—C10—C11	121.3 (3)
H16A—C16—H16B	109.5	C9—C10—Br2	118.1 (3)
C15—C16—H16C	109.5	C11—C10—Br2	120.6 (3)
H16A—C16—H16C	109.5	C7—C12—C11	118.2 (3)
H16B—C16—H16C	109.5	C7—C12—H12	120.9
C2—C1—C6	119.4 (3)	C11—C12—H12	120.9
C2—C1—H1	120.3	C5—N—C8	108.6 (3)
C6—C1—H1	120.3	C5—N—C13	125.1 (3)
C1—C2—C3	119.9 (3)	C8—N—C13	126.2 (3)
C1—C2—Br1	118.0 (3)		
			
N—C13—C14—C15	−62.0 (4)	C12—C7—C8—N	178.9 (3)
C13—C14—C15—C16	179.9 (3)	C6—C7—C8—N	−0.2 (4)
C6—C1—C2—C3	1.6 (5)	N—C8—C9—C10	−178.2 (3)
C6—C1—C2—Br1	−177.2 (2)	C7—C8—C9—C10	0.1 (5)
C1—C2—C3—C4	−2.2 (5)	C8—C9—C10—C11	0.0 (5)
Br1—C2—C3—C4	176.5 (3)	C8—C9—C10—Br2	176.4 (3)
C1—C2—C3—Br3	176.5 (3)	C12—C11—C10—C9	−0.4 (5)
Br1—C2—C3—Br3	−4.7 (4)	Br4—C11—C10—C9	178.5 (3)
C2—C3—C4—C5	0.5 (5)	C12—C11—C10—Br2	−176.7 (3)
Br3—C3—C4—C5	−178.3 (2)	Br4—C11—C10—Br2	2.3 (4)
C3—C4—C5—N	−179.3 (3)	C8—C7—C12—C11	−0.7 (5)
C3—C4—C5—C6	1.7 (5)	C6—C7—C12—C11	178.0 (4)
C2—C1—C6—C5	0.6 (5)	C10—C11—C12—C7	0.8 (5)
C2—C1—C6—C7	−179.7 (4)	Br4—C11—C12—C7	−178.2 (2)
N—C5—C6—C1	178.6 (3)	C4—C5—N—C8	−177.9 (3)
C4—C5—C6—C1	−2.3 (5)	C6—C5—N—C8	1.1 (4)
N—C5—C6—C7	−1.2 (4)	C4—C5—N—C13	1.3 (6)
C4—C5—C6—C7	177.9 (3)	C6—C5—N—C13	−179.6 (3)
C1—C6—C7—C12	2.2 (7)	C9—C8—N—C5	177.8 (3)
C5—C6—C7—C12	−178.0 (4)	C7—C8—N—C5	−0.6 (4)
C1—C6—C7—C8	−178.9 (4)	C9—C8—N—C13	−1.4 (6)
C5—C6—C7—C8	0.8 (4)	C7—C8—N—C13	−179.8 (3)
C12—C7—C8—C9	0.3 (5)	C14—C13—N—C5	−81.3 (4)
C6—C7—C8—C9	−178.7 (3)	C14—C13—N—C8	97.8 (4)
